# Mitotic spindle: kinetochore fibers hold on tight to interpolar bundles

**DOI:** 10.1007/s00249-017-1244-4

**Published:** 2017-07-19

**Authors:** Iva M. Tolić

**Affiliations:** Division of Molecular Biology, Ruđer Bošković Institute, Bijenička cesta 54, 10000 Zagreb, Croatia

**Keywords:** Mitosis, Kinetochore microtubules, k-fibers, Bridging fibers, Overlap microtubules, Kinetochore, Metaphase, Crosslinkers, PRC1, Motor proteins, Kinesin

## Abstract

When a cell starts to divide, it forms a spindle, a micro-machine made of microtubules, which separates the duplicated chromosomes. The attachment of microtubules to chromosomes is mediated by kinetochores, protein complexes on the chromosome. Spindle microtubules can be divided into three major classes: kinetochore microtubules, which form k-fibers ending at the kinetochore; interpolar microtubules, which extend from the opposite sides of the spindle and interact in the middle; and astral microtubules, which extend towards the cell cortex. Recent work in human cells has shown a close relationship between interpolar and kinetochore microtubules, where interpolar bundles are attached laterally to kinetochore fibers almost all along their length, acting as a bridge between sister k-fibers. Most of the interpolar bundles are attached to a pair of sister kinetochore fibers and vice versa. Thus, the spindle is made of modules consisting of a pair of sister kinetochore fibers and a bundle of interpolar microtubules that connects them. These interpolar bundles, termed bridging fibers, balance the forces acting at kinetochores and support the rounded shape of the spindle during metaphase. This review discusses the structure, function, and formation of kinetochore fibers and interpolar bundles, with an emphasis on how they interact. Their connections have an impact on the force balance in the spindle and on chromosome movement during mitosis because the forces in interpolar bundles are transmitted to kinetochore fibers and hence to kinetochores through these connections.

## Introduction

**Fig. 1:**
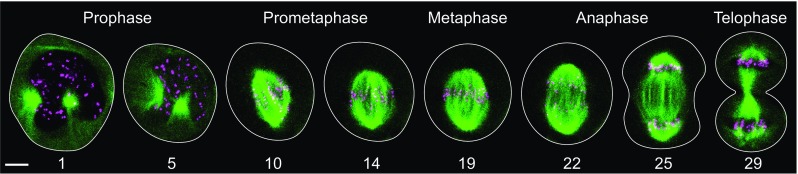
Mitosis in a human cell. Microtubules are shown in *green* and kinetochores in *magenta*, in a U2OS cell expressing CENP-A-GFP and mCherry-α-tubulin. The *white line* marks the cell outline; time is given in minutes; *scale bar* represents 5 µm

Cell division is one of the most fundamental processes in the living world. At the onset of division the cell assembles a spindle (Fig. 1), a fascinating and complex micro-machine made of microtubules and the accompanying proteins (McIntosh et al. 2012; Pavin and Tolic 2016; Prosser and Pelletier 2017). Spindle microtubules attach to chromosomes via kinetochores, protein complexes assembled on the centromeres of each chromosome (Musacchio and Desai 2017). The central question in the field is how the cell achieves accurate chromosome segregation through interactions between kinetochores, microtubules, and the associated proteins.

**Fig. 2:**
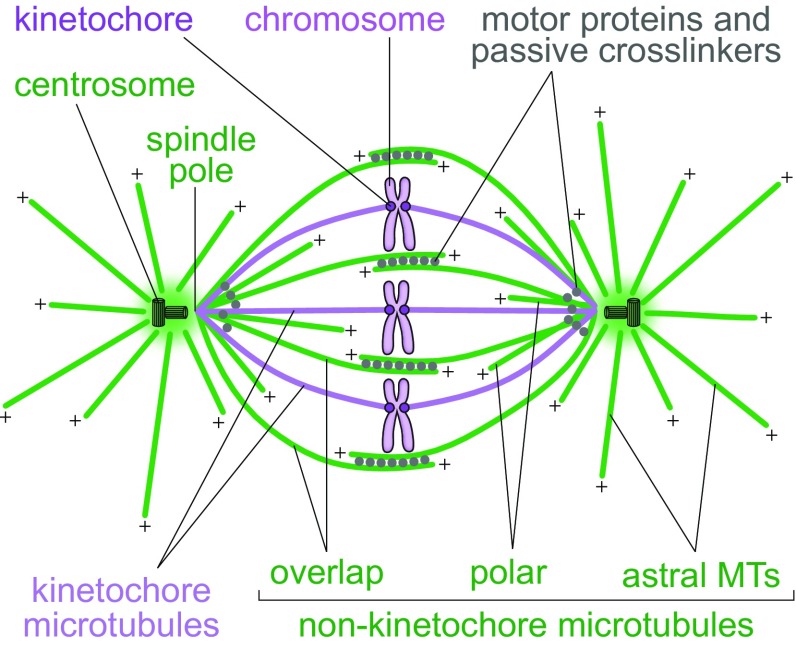
Textbook picture of the spindle. Redrawn and modified from (Alberts et al. 2014)

Spindles in mammalian cells contain hundreds of microtubules, which are connected in a complex fashion with the help of various microtubule-associated proteins including motor proteins and passive crosslinkers. To help understand how the spindle functions, a large amount of experimental data has been condensed into a simplified picture, in which the microtubules are divided into several categories (Fig. 2). First, one can divide the microtubules with respect to whether they end at the kinetochore or not. Kinetochore microtubules end at the kinetochore and form parallel bundles known as kinetochore fibers or k-fibers. Non-kinetochore microtubules can be found as single microtubules, in parallel bundles, or in antiparallel bundles. When divided with respect to their location, non-kinetochore microtubules include those that grow from the spindle pole towards the cell cortex, known as astral microtubules, those that grow towards the spindle equator and have free ends, which are often called polar, and those that form antiparallel overlaps in the central part of the spindle, known as interpolar or overlap microtubules (Alberts et al. 2014).

This review is focused on kinetochore fibers and interpolar bundles, with an emphasis on the relationship between them in the context of human somatic cells. Even though these bundles are generally thought of as being physically separate in the region close to kinetochores, recent work suggests that they are tightly connected. This coupling has important implications for the forces acting on kinetochores and thus for the process of cell division.

## Kinetochore fibers

Kinetochore fibers are the main generators of forces that move the chromosomes during mitosis. When sister kinetochores become attached to microtubule fibers extending from the opposite spindle poles, which is known as biorientation, these fibers hold the chromosomes near the spindle equator (Maiato et al. 2017). Their stable interaction with kinetochores is required for the cell to pass the spindle assembly checkpoint and proceed from metaphase into anaphase (Musacchio 2015). During anaphase, kinetochore fibers shorten by depolymerization at the kinetochore and at the pole, thereby segregating sister chromatids towards the opposite spindle poles (Asbury 2017).

**Fig. 3:**
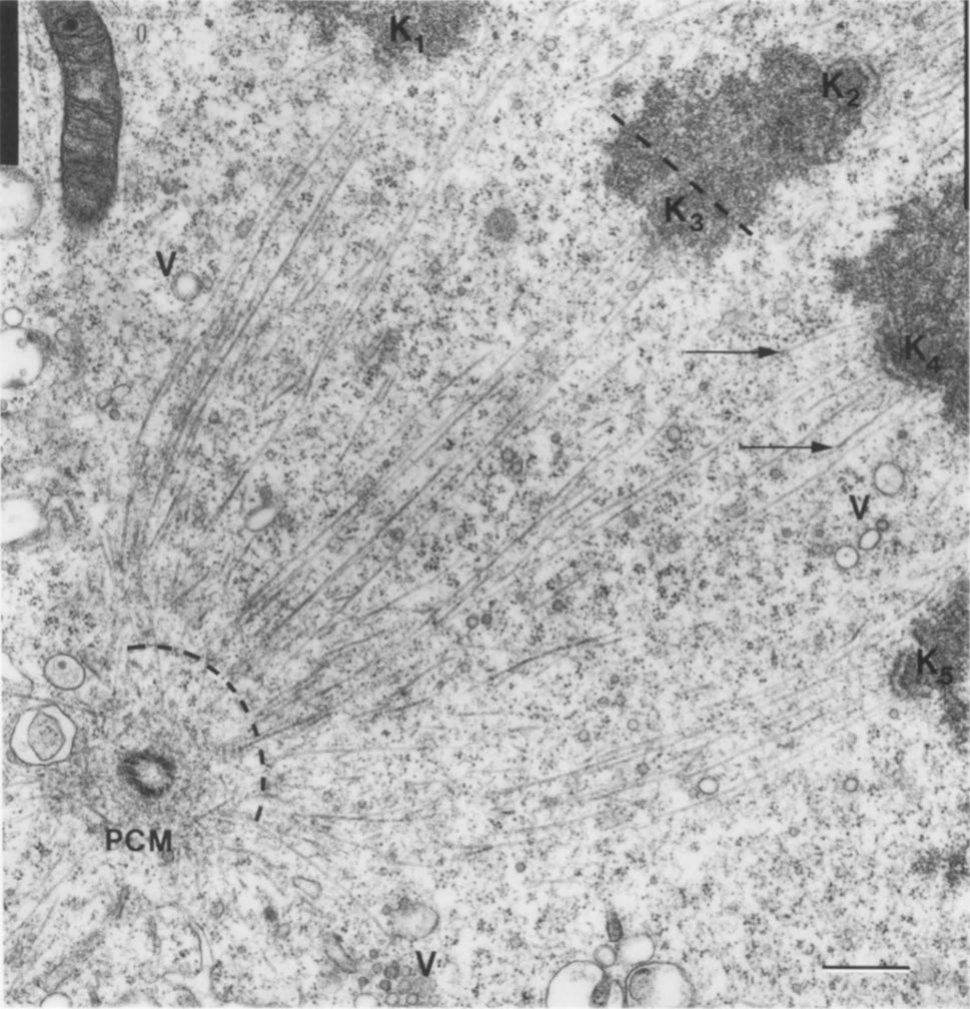
Kinetochore fibers. Electron micrograph of a metaphase spindle in a PtK1 cell. Kinetochore microtubules are visible as *thin lines* extending between the boundary of the spindle pole (*curved dashed line*) and the kinetochores (K_1_–K_5_). *Arrows* mark microtubules that leave the plane of section; *V* vesicles, *PCM* pericentriolar material; *scale bar* 0.5 µm. Image reproduced with permission from (McDonald et al. 1992)

Most of the existing information about kinetochore fiber structure has been obtained by electron microscopy studies (Fig. 3). The cross-section of a metaphase kinetochore fiber in PtK1 cells consists of 20–30 microtubules (McDonald et al. 1992; McEwen et al. 1997; Brinkley and Cartwright 1971; McIntosh et al. 1975). The number of microtubules in a kinetochore fiber increases as mitosis progresses and the fiber matures. At late prometaphase, the average number of microtubules on fully congressed kinetochores is 20, at late metaphase 24, and at early anaphase 28 (McEwen et al. 1997). In HeLa cells, a metaphase kinetochore fiber consists of 17 microtubules on average (McEwen et al. 2001; Wendell et al. 1993). The majority of the microtubules in a kinetochore fiber in PtK1 cells extend between the kinetochore and the spindle pole (Rieder 1981; McDonald et al. 1992; Brinkley and Cartwright 1971). The minus ends of these microtubules are found at the border of the spindle pole, roughly 0.4–0.5 µm away from the centrioles (McDonald et al. 1992).

Kinetochore fibers are the most stable bundles in the metaphase spindle, given that interpolar and astral microtubules disassemble after perturbations such as cold treatment, while kinetochore microtubules are more resistant (Brinkley and Cartwright 1975; Rieder 1981). The microtubules in a kinetochore fiber are linked by clathrin in a complex with the transforming acidic coiled-coil protein 3 (TACC3) and colonic, hepatic tumor overexpressed gene (ch-TOG). Clathrin is required for kinetochore fiber stability and proper chromosome congression to the metaphase plate (Booth et al. 2011; Royle et al. 2005). These proteins are part of a network of microtubule connectors, called the mesh (Nixon et al. 2015), which most likely pull kinetochore microtubules together and provide structural integrity to the kinetochore fiber.

During metaphase, kinetochore fibers show remarkable dynamic behavior. At the kinetochore end, they exhibit switching between growth and shrinkage, with a net growth over time (Mitchison et al. 1986; Rieder and Salmon 1998). This growth is accompanied by the disassembly at the spindle pole, resulting in a flux of the entire kinetochore fiber poleward (Mitchison et al. 1986; Mitchison 1989). Even though kinetochore fibers grow and shrink as a unit, the growth and shrinkage of individual microtubules in a single fiber is not necessarily synchronized. Electron tomography has revealed that plus ends of roughly 70% of kinetochore microtubules have curved protofilaments (VandenBeldt et al. 2006), suggesting that these microtubules are in a depolymerizing state based on in vitro observations that protofilaments of depolymerizing microtubules coil inside out (Mandelkow et al. 1991). Yet, this interpretation should be taken with caution because in vivo protofilaments can bend outwards even when the microtubule is growing (Kukulski et al. 2011; Hoog et al. 2007; McIntosh et al. 2013). Live-cell imaging of EB3, a protein that tracks growing plus ends of microtubules, showed that a kinetochore fiber consists of a mixture of polymerizing and depolymerizing microtubules, with a small polymerization bias for fibers that exhibit net growth (Armond et al. 2015). Thus, the dynamics of individual microtubules within a kinetochore fiber is not coordinated, but this does not prevent the dynamic behavior of the fiber as a unit.

**Fig. 4:**
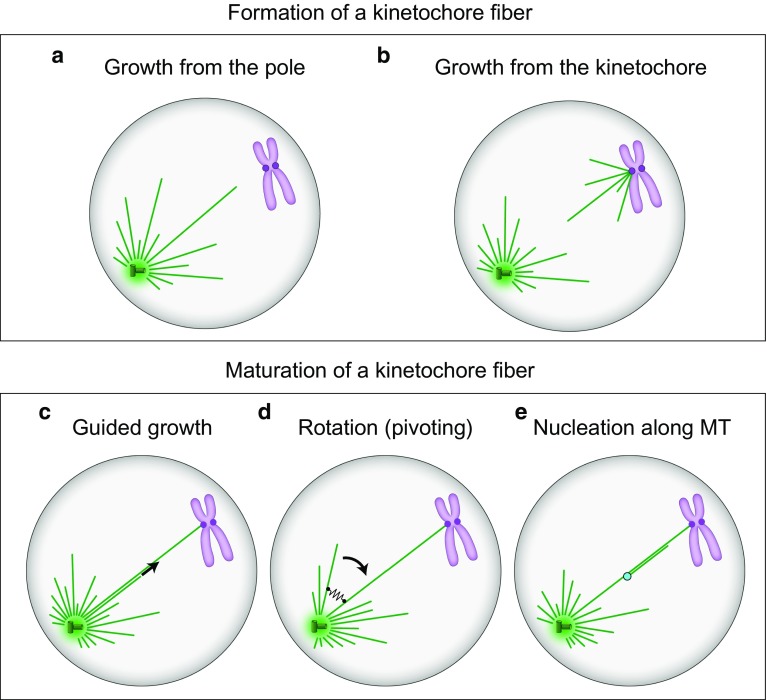
Formation (**a**, **b**) and maturation (**c**–**e**) of kinetochore fibers. **a**, **b** Microtubules of the future kinetochore fiber are formed at the spindle pole or at the kinetochore, respectively. **c** New microtubules are formed at the pole and grow along the existing kinetochore microtubules. **d** New microtubules are formed at the pole and grow at an angle with respect to the existing microtubules, forming a V-shape. They rotate around the spindle pole and eventually approach the existing kinetochore microtubules, which is followed by their binding via crosslinking proteins (*black spring*). **e** New microtubules are nucleated at the nucleation sites (*light blue*) along the existing kinetochore fiber. In all panels, microtubules are shown as *green lines*, centrosomes as *green circles* with *small cylinders* representing centrioles inside, and chromosomes are *purple* with kinetochores depicted as *dark purple circles*

How kinetochore fibers form in human cells is an open question. In principle, growth of the first microtubules of a nascent kinetochore fiber may be initiated at the spindle pole (Fig. 4a) or at the kinetochore (Fig. 4b) (Rieder 2005). If they grow from the spindle pole, they have to capture the kinetochores. A pioneering idea concerning the capture process is based on microtubule dynamics (Kirschner and Mitchison 1986; Holy and Leibler 1994). In this scenario, known as search-and-capture, microtubules grow in random directions from the spindle pole. If a microtubule does not interact with a kinetochore, it undergoes catastrophe and shrinks back to the pole. New microtubules grow, each of them having a chance to reach a kinetochore. A microtubule that eventually captures a kinetochore becomes stabilized by this interaction, thereby establishing the basis of a kinetochore fiber. Several mechanisms may accelerate this process (Wollman et al. 2005; Paul et al. 2009; Magidson et al. 2011, 2015) and have been reviewed elsewhere (Mogilner and Craig 2010; Pavin and Tolic-Norrelykke 2014; Heald and Khodjakov 2015; Pavin and Tolic 2016; Prosser and Pelletier 2017).

Alternatively, growth of kinetochore microtubules may be initiated at the kinetochore (Fig. 4b). Microtubule growth from kinetochores has been seen on isolated human mitotic chromosomes (Telzer et al. 1975; McGill and Brinkley 1975) and in mammalian cells recovering from treatments with microtubule inhibitors (Witt et al. 1980; De Brabander et al. 1981). Initiation of kinetochore fiber formation at the kinetochore has also been observed in untreated cells (Khodjakov et al. 2003; Maiato et al. 2004). However, these events are rare and do not represent a dominant mechanism of kinetochore fiber formation in mammalian somatic cells.

A mature kinetochore fiber consists of about 20 parallel microtubules. This thick fiber is formed most likely by stepwise addition of new microtubules to the immature fiber. These new microtubules required for the maturation of the kinetochore fiber may grow from the spindle pole region along the existing fiber (Fig. 4c). A recent study supports this scenario by showing that kinesin-14 motors are recruited to the plus end of a microtubule and guide its growth along another microtubule (Molodtsov et al. 2016).

New microtubules growing from the spindle pole, which may eventually become part of an existing kinetochore fiber, do not necessarily grow along this fiber. Instead, they may grow at an angle with respect to the existing fiber, forming a V-shape with it (Fig. 4d). These V-shaped configurations are often seen in yeasts, where microtubules grow from the spindle pole body, a centrosome equivalent in yeast, in different directions (Sagolla et al. 2003). Interestingly, these microtubules rotate about the pivot at the spindle pole body (Kalinina et al. 2013; Baumgartner and Tolic 2014; Cojoc et al. 2016a). The concept of microtubule rotation, which is also known as pivoting, swiveling, or angular motion, may be important for the formation of kinetochore fibers because this motion helps the microtubules as they search for targets such as kinetochores, cortical anchors, or other microtubules (Pavin and Tolic-Norrelykke 2014). Pivoting is the dominant mechanism by which microtubules search for kinetochores during mitosis in fission yeast (Kalinina et al. 2013). Microtubule pivoting also contributes to kinetochore capture at the onset of meiosis I in the same organism (Cojoc et al. 2016a). Modeling work suggests that if the microtubules undergo both dynamic instability and rotation, the relative contribution of the dynamic instability versus rotation is higher for more dynamic microtubules (Cojoc et al. 2016a; Blackwell et al. 2017b). In *Drosophila* S2 cells, microtubules extending from the kinetochore are initially not oriented towards a spindle pole, but they pivot around the kinetochore while growing and eventually become captured by the microtubules growing from the spindle pole (Maiato et al. 2004). Microtubule pivoting also helps astral microtubules to find cortical anchor sites required for the movement of the spindle from the mother cell into the bud in budding yeast (Baumgartner and Tolic 2014). In all these cases, microtubule pivoting allows them to swipe through space, which increases the explored volume and makes the search process more efficient (Kalinina et al. 2013; Pavin and Tolic-Norrelykke 2014).

Pivoting of microtubules may be important for the transformation of a V-shaped microtubule structure into a parallel bundle, which may be relevant for the formation of kinetochore fibers (Fig. 4d). We have recently introduced a pivot-and-bond model, in which microtubules pivot around the spindle pole and bond with each other by crosslinking proteins (Prelogović et al. 2017). Our experiments in fission yeast show that microtubules pivot around the spindle pole before getting aligned and forming a parallel bundle, and that bundle formation relies to a large extent on the crosslinker anaphase spindle elongation protein (Ase1), a homolog of the human protein regulator of cytokinesis 1 (PRC1) (Prelogović et al. 2017). Thus, in the pivot-and-bond model, microtubules explore the space by performing rotational diffusion and ultimately approach one another, which in turn allows the crosslinking proteins to connect the microtubules into a stable parallel bundle. Likewise, this mechanism may contribute to the formation and maturation of kinetochore fibers in human cells.

Finally, microtubules needed for the maturation of a kinetochore fiber may be formed at locations other than the spindle pole (Fig. 4e). For example, some microtubules are nucleated at sites along the preexisting mother microtubule (Murata et al. 2005; Goshima et al. 2008; Mahoney et al. 2006). In meiotic *Xenopus* egg extracts, these new microtubules grow at small angles and with the same polarity as the mother microtubule, which makes them suitable to generate parallel microtubule bundles such as kinetochore fibers (Petry et al. 2013). This mechanism may be at work also in human somatic cells.

## Interpolar microtubule bundles

Interpolar bundles are important for the structural integrity of the spindle during its formation in prometaphase (Tanenbaum and Medema 2010). When the cell enters anaphase, interpolar bundles start to elongate, which results in pole separation and spindle elongation (Scholey et al. 2016). Because kinetochore fibers, which are attached to chromosomes, are mechanically connected to the pole either directly or indirectly via connections to other pole-bound microtubules, the separation of the spindle poles contributes to chromosome segregation.

**Fig. 5:**
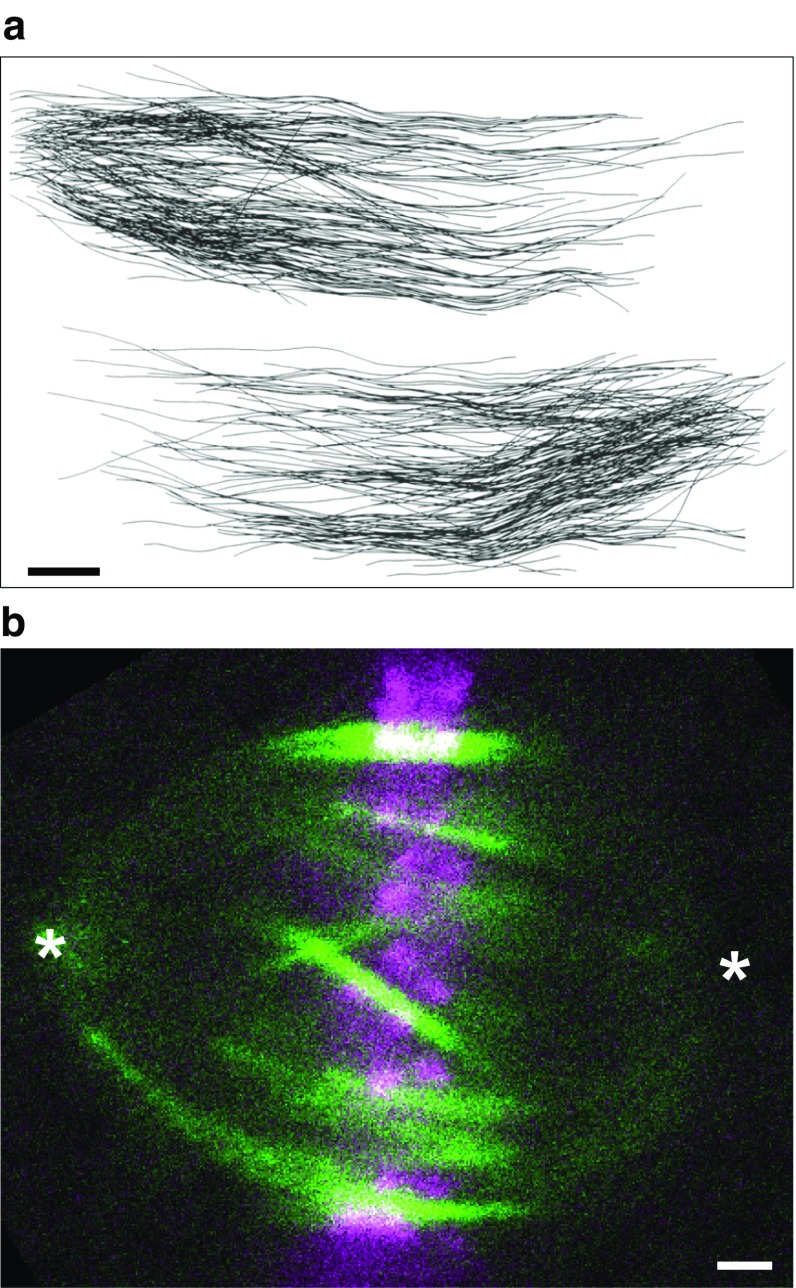
Interpolar bundles. **a** Interpolar microtubules in a PtK1 cell in early anaphase, reconstructed from an electron micrograph. Microtubules whose minus ends were associated with the pole on the *left* or the *right* are shown separately. *Scale bar* 1 µm. Image reproduced with permission from (Mastronarde et al. 1993). **b** Interpolar bundles in a HeLa cell visualized by PRC1-GFP (*green*), which binds to antiparallel microtubule overlaps. The antiparallel overlap zones are ~5 µm long and found in the central part of the spindle. Kinetochores (mRFP-CENP-B) are visible in *magenta*. *Asterisks* mark the spindle poles; *scale bar* 1 µm. Image reproduced with permission from (Polak et al. 2017)

As in the case of kinetochore fibers, the structure and spatial distribution of interpolar bundles has been revealed by electron microscopy (Fig. 5a). In PtK1 cells, the minus ends of interpolar microtubules are found throughout the spindle (Mastronarde et al. 1993), in contrast to the kinetochore microtubules whose minus ends are mostly near the poles (McDonald et al. 1992). Interpolar microtubules meet in the equatorial region of the spindle, where they form antiparallel overlaps (Brinkley and Cartwright 1971). In addition, a substantial fraction of non-kinetochore microtubules end before they reach the spindle equator (McIntosh et al. 1975).

The major passive crosslinker of antiparallel interpolar microtubules is PRC1/Ase1 (Pellman et al. 1995; Mollinari et al. 2002; Bieling et al. 2010; Jiang et al. 1998). Thus, PRC1 can be used to visualize the overlap zones of interpolar microtubules throughout the spindle (Fig. 5b). During metaphase, both endogenous and GFP-tagged PRC1 is found in the central part of the spindle, in streaks running roughly parallel to the spindle axis (Polak et al. 2017). The PRC1 streaks are about 5 µm long, reflecting the length of the antiparallel overlaps. Electron micrographs revealed overlap zones of a similar length (Mastronarde et al. 1993). These overlaps become shorter in late anaphase, which is visible both in electron micrographs (Mastronarde et al. 1993) and as accumulation of PRC1 in short bands in the spindle midzone (Jiang et al. 1998; Mollinari et al. 2002).

Microtubules that form antiparallel overlaps can slide with respect to one another. Early studies on diatom and fission yeast spindles have shown that sliding of interpolar microtubules, which is powered by motor proteins, is the key mechanochemical process driving anaphase spindle elongation (Cande and McDonald 1985; Masuda et al. 1990). For metaphase spindles, early work on budding yeast has established that counteracting forces produced by oppositely oriented motors maintain the spindle structure (Saunders and Hoyt 1992). In particular, kinesin-5 motors Cut7/Cin8/Eg5/KIF11 slide the microtubules, and thus the spindle poles, apart by walking along the microtubules away from the pole, i.e., towards the plus end of the microtubules (Hagan and Yanagida 1990; Le Guellec et al. 1991; Sawin et al. 1992; Kapitein et al. 2005; Saunders and Hoyt 1992). Other motors pull the spindle poles towards each other by walking along the microtubules towards the pole, i.e., towards the minus end of the microtubule, such as kinesin-14 motors Ncd/HSET/KifC1 (Endow et al. 1990; McDonald et al. 1990; Cai et al. 2009) and dynein (Tanenbaum et al. 2008, 2013). Interestingly, kinesin-5 motors can also move towards the minus end of the microtubules, when walking on a single microtubule or in a non-crowded environment on antiparallel microtubules (Roostalu et al. 2011; Edamatsu 2014; Britto et al. 2016). Similarly, kinesin-14 can reverse its direction of movement from the minus end toward the plus end under a low external force (Molodtsov et al. 2016). Coordination of the forces exerted by motor proteins, together with the forces arising from microtubule dynamics, are thought to be responsible for the maintenance of a constant spindle length during metaphase and for spindle elongation in anaphase (Brust-Mascher et al. 2004; Goshima and Scholey 2010; Sharp et al. 1999; Scholey et al. 2016; Saunders and Hoyt 1992).

**Fig. 6:**
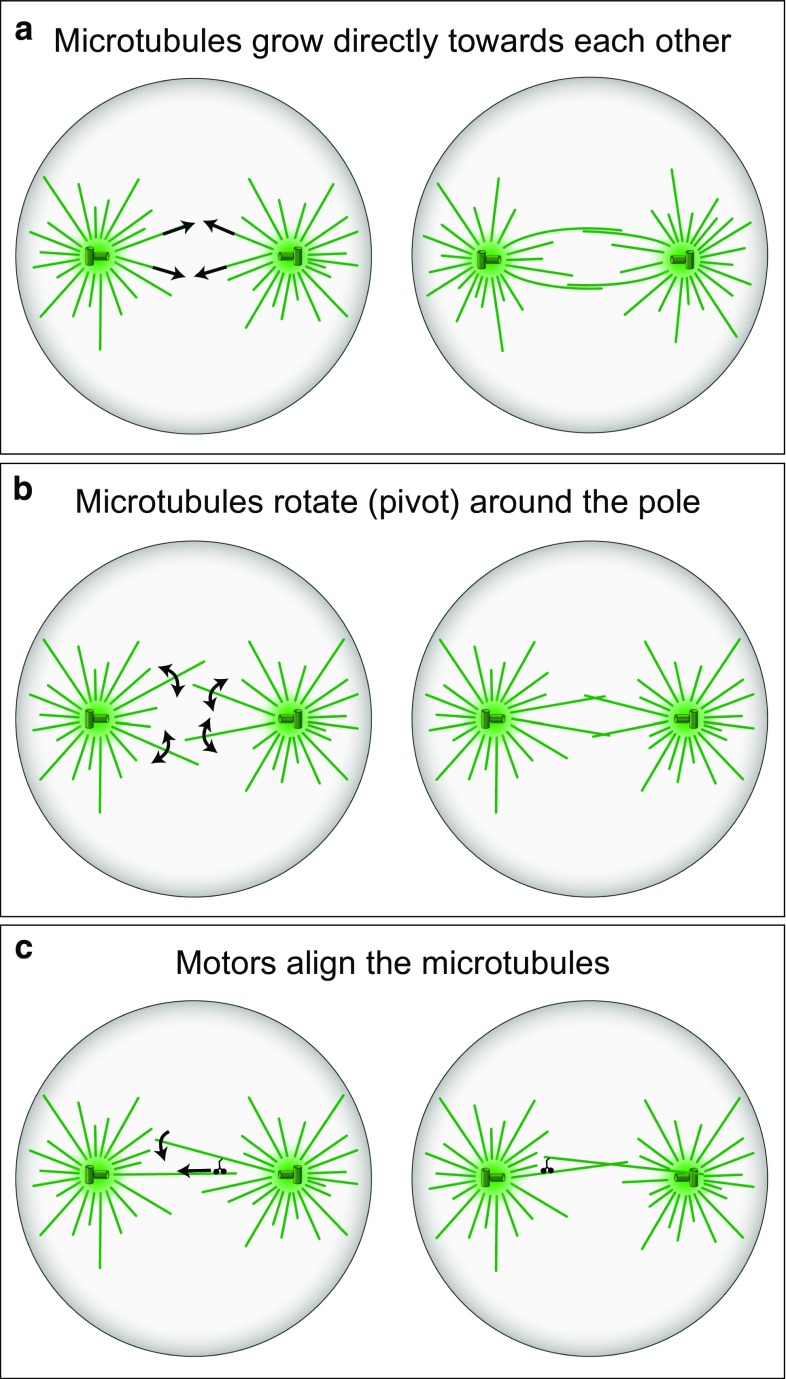
Formation of interpolar microtubule bundles. **a** Microtubules grow from the two spindle poles in arbitrary directions (*arrows*), eventually approach each other and form interpolar bundles with the help of crosslinking proteins. **b** Microtubules rotate around the spindle pole (*arrows*), which allows them to explore the space more efficiently and approach the microtubules extending from the opposite pole more quickly. **c** Microtubules extending from the opposite poles are connected by minus end directed motors (*small black cherry-like object*), which align the two microtubules (*curved arrow*) as they walk towards the spindle pole (*straight arrow*). In all panels, microtubules are shown as *green lines*, and centrosomes as *green circles* with *small cylinder*s representing centrioles

As in the case of kinetochore fibers, the process of formation of interpolar bundles in human cells is not well understood. Presumably, interpolar bundles form during early mitosis through the interactions of microtubules growing from the opposite spindle poles. In the search-and-capture picture (Kirschner and Mitchison 1986), as discussed above, microtubules grow in random directions from the spindle poles (Fig. 6a). Because of this dynamic behavior, microtubules sometimes get close to those extending from the other spindle pole. In the region where such microtubules meet, they become crosslinked by specific crosslinkers and motor proteins, which stabilizes the microtubules against shrinking. Formation of interpolar bundles through these processes has been explored by computer simulations (Nédélec 2002).

The swiping motion of microtubules during their rotation around the spindle pole, which was found in yeasts (Kalinina et al. 2013; Baumgartner and Tolic 2014; Cojoc et al. 2016a; Pavin and Tolic-Norrelykke 2014), may help the microtubules extending from the two poles to find each other much faster than in the case without rotation (Fig. 6b). A recent study incorporated microtubule rotation around the spindle pole body into a model of spindle formation in fission yeast (Blackwell et al. 2017a). Computer simulations of that model suggest that a decreased microtubule rotation results in shorter spindles and fewer microtubules in the bundle connecting the two spindle poles. Thus, proper spindle assembly requires the connections between the microtubules and the spindle pole body to be strong to keep them linked and at the same time flexible to allow free rotation of microtubules.

Microtubule rotation may occur not only before the microtubules from the opposite spindle poles meet, but also after their initial interaction. Indeed, during the formation of interpolar bundles, microtubules that meet at an oblique angle must rotate to get aligned in an antiparallel fashion. A study of spindle assembly in budding yeast showed that cells lacking kinesin-14 motors have poorly aligned interpolar microtubules (Hepperla et al. 2014). This finding was explained by a model in which microtubules growing from the two spindle poles become connected by kinesin-14 motors. The motors walk along one microtubule towards its minus end, while being bound to other microtubule, thereby aligning the two microtubules into an interpolar bundle (Fig. 6c). Thus, in this model, motor-driven angular motion of microtubules results in their alignment and formation of an antiparallel bundle (Hepperla et al. 2014). Even though the concept of angular motion of microtubules comes from studies on yeast cells, it may also be relevant for the formation of interpolar bundles in human cells.

## Connections between kinetochore fibers and interpolar microtubules

A textbook picture of the spindle depicts kinetochore fibers and interpolar bundles as distinct clusters, physically separated from each other along their length except in the regions near the spindle poles (Fig. 2). The spindle is evidently simplified in this picture, lacking a large part of the complex spatial distribution and intricate connections between its building blocks. Nevertheless, the current view in the field is that kinetochore fibers and interpolar bundles are largely independent structures in the region close to kinetochores.

**Fig. 7:**
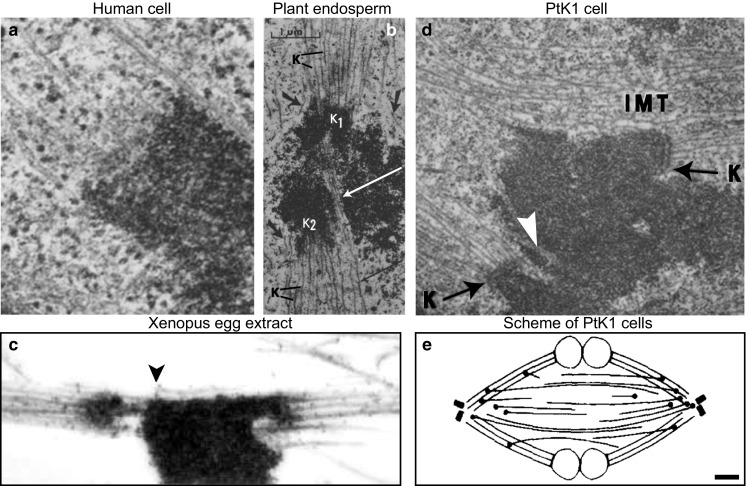
Interpolar microtubules are found in the vicinity of kinetochore microtubules and kinetochores during metaphase. Example images of metaphase spindles from different organisms, obtained by using electron microscopy, are shown. **a** Area around a kinetochore in a human WI-38 cell. Several microtubules end at the kinetochore, whereas a non-kinetochore microtubule passes the kinetochore zone. Image reproduced with permission from (McIntosh and Landis 1971). **b** Area around kinetochores in the spindle of *Haemanthus katherinae* endosperm. Two kinetochores (K_1_ and K_2_) are attached to kinetochore microtubules (K). Non-kinetochore microtubules (*long arrows*) intermingle with kinetochore microtubules. Image reproduced with permission from (Jensen 1982). **c** Area around kinetochores in a *Xenopus* egg extract spindle. Kinetochore fibers are associated with microtubules that do not end at kinetochores (*arrowhead*). Image reproduced with permission from (Ohi et al. 2003). **d** Area around kinetochores in a PtK1 cell. Interpolar microtubules (IMT) and microtubules which penetrate the chromosome (*white arrow*) are found near the kinetochores (K). Magnification ×21,000; image reproduced with permission from (Brinkley and Cartwright 1971). **e** Arrangement of interpolar and kinetochore microtubules in a metaphase PtK1 spindle, based on reconstructions of microtubules from electron micrographs. *Black circles* mark the minus ends of interpolar microtubules, which are found farther from the pole than those of kinetochore microtubules and most are in or near kinetochore fibers. *Scale bar* 1 µm; image reproduced with permission from (Mastronarde et al. 1993)

Remarkably, several studies on cells from various species have shown that different microtubule bundles in the spindle are in close contact. Electron micrographs have revealed non-kinetochore microtubules that extend along the kinetochore fiber, pass the kinetochore and enter the region between sister kinetochores, in metaphase spindles of human WI-38 cells (Fig. 7a) (McIntosh and Landis 1971) and HeLa cells (Nixon et al. 2017), plant endosperm (Fig. 7b) (Jensen 1982), and Xenopus egg extracts (Fig. 7c) (Ohi et al. 2003). Intermixing of non-kinetochore microtubules with kinetochore fibers has been seen in the region close to the kinetochore in PtK1 cells (Fig. 7d) (McDonald et al. 1992; Brinkley and Cartwright 1971). The minus ends of interpolar microtubules were found to be clustered in the bundles of kinetochore microtubules (Fig. 7e) (Mastronarde et al. 1993). In fission yeast and budding yeast, interpolar microtubules form a single bundle, while kinetochore microtubules lie next to it (Ding et al. 1993; Winey et al. 1995). Thus, electron microscopy has revealed that non-kinetochore microtubules can be found in the neighborhood of kinetochore microtubules in various cell types, which implies that the microtubules from these two groups may be physically linked. The existence of such links may be important for the force balance in the spindle and chromosome movement because the forces generated by non-kinetochore microtubules, in particular by the interpolar bundles, may be transmitted to kinetochore fibers and hence to kinetochores through these connections.

Connections between different fibers in the metaphase spindle have been included in physical models. In one model, kinetochore fibers and interpolar microtubules are coupled by viscoelastic links (Matos et al. 2009). This model suggests that the poleward flux of spindle microtubules is able to equalize the tension over all kinetochores, ensuring coordinated segregation of chromosomes in anaphase (Pereira and Maiato 2012; Matos et al. 2009). In another model, neighboring kinetochore fibers are connected by elastic springs. This model was used to explain the observation that neighboring kinetochore pairs oscillate in a coordinated manner (Vladimirou et al. 2013). Thus, both models indicate that connections between neighboring fibers can synchronize the dynamics of different kinetochore pairs during metaphase.

In anaphase, the current understanding of forces acting on chromosomes is based on events occurring at the ends of the kinetochore fiber. These forces are associated with shortening of kinetochore microtubules at the kinetochore and at the spindle pole (Maiato and Lince-Faria 2010; Asbury 2017). In addition, forces can be generated at the kinetochore fiber end created by laser cutting, where dynein is recruited and drives poleward movement of the end of the kinetochore fiber by walking along neighboring pole-anchored microtubules (Elting et al. 2014; Sikirzhytski et al. 2014). Yet, early studies suggested that forces act along the length of the kinetochore fiber rather than only at its ends (Ostergren 1951). These forces may be generated by the sliding of antiparallel interpolar microtubules over one another (McIntosh et al. 1969) and transmitted to kinetochore fibers through the crosslinks between interpolar and kinetochore microtubules (Margolis et al. 1978; Goode 1981; Mitchison 2005). Thus, forces acting along the lattice of kinetochore fiber microtubules may contribute to the movement of chromosomes in anaphase. However, these ideas have not been directly tested so far.

## Interpolar microtubules act as a bridge between sister kinetochore fibers

**Fig. 8:**
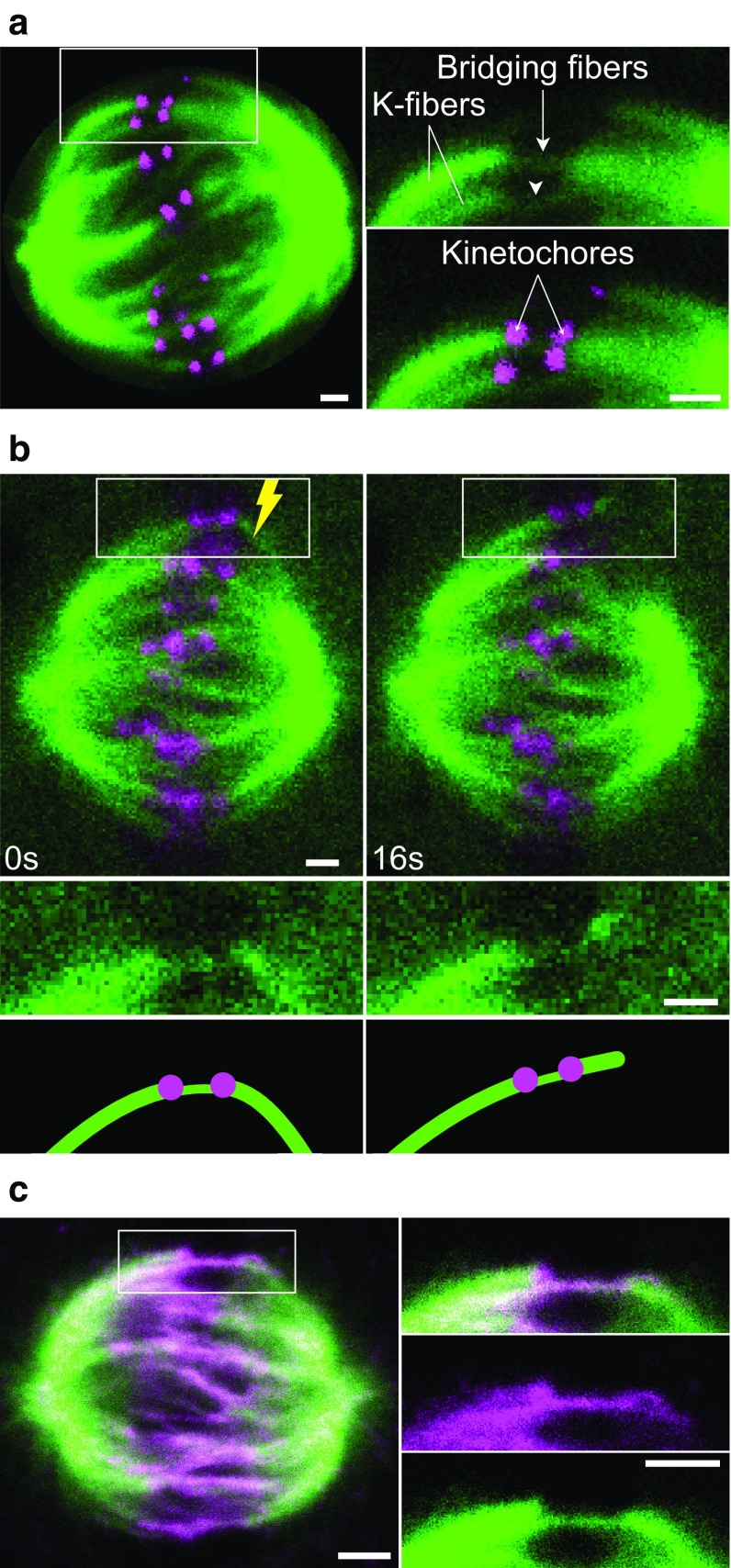
Bridging microtubules link sister kinetochore fibers. **a** Spindle in a HeLa cell with microtubules shown in green (stained with SiR-tubulin) and kinetochores in magenta (EGFP-CENP-A). Enlargements of the *boxed region* (*top* tubulin, *bottom* merge) show bridging fibers connecting sister kinetochore fibers (k-fibers). Compared with kinetochore fibers, bridging fibers contain fewer microtubules and hence are less bright. **b** Laser ablation of a kinetochore fiber in a HeLa cell with microtubules shown in green (tubulin-GFP) and kinetochores in magenta (mRFP-CENP-B). Time-lapse images of the spindle (*top*) and enlargements of the *boxed region* (*middle* tubulin, *bottom* schemes) are shown. After the cut (*yellow lightning sign*), the bridging fiber moved together with sister kinetochores and their fibers in the direction away from the spindle. Image reproduced with permission from (Kajtez et al. 2016). **c** Spindle in a HeLa cell with microtubules shown in green (tubulin-GFP) and endogenous PRC1 in magenta (immunostained, Alexa Fluor-555 labeled). Enlargements of the boxed region (*top* merge, middle: PRC1, *bottom* tubulin). The PRC1 signal is found in the central part of the bridging fiber, extending ~2 µm poleward from each kinetochore. Image reproduced with permission from (Polak et al. 2017). *Scale bars* in all panels are 1 µm

We have recently explored the relationship between interpolar and kinetochore fibers (Kajtez et al. 2016). By using fluorescence microscopy images of live human cells in metaphase, we observed that a bundle of interpolar microtubules connects a pair of kinetochore fibers bound to sister kinetochores (Fig. 8a). This interpolar bundle looks like a bridge between sister kinetochore fibers, hence we called it *bridging fiber*. It is important to note that the bridging fiber is defined by its function, as a fiber that links two sister kinetochore fibers.

The non-kinetochore microtubules found in the vicinity of kinetochores, illustrated in Fig. 7, may be bridging microtubules. However, images such as those shown in Figs. 7 and 8a cannot distinguish whether these microtubules are physically linked with kinetochore fibers or they just happened to lie close to them. Thus, to test the interaction between different microtubules in the spindle, it was crucial to develop an assay that can identify such interactions. We used laser ablation to sever a kinetochore fiber in HeLa, U2OS and PtK1 cells (Buda et al. 2017; Kajtez et al. 2016; Milas and Tolic 2016), similar to previous cutting experiments (Cojoc et al. 2016b; Elting et al. 2014; Sikirzhytski et al. 2014). We reasoned that if the bridging fiber is physically linked with kinetochore fibers, they are expected to move together after the severing. Indeed, we observed that the bridging fiber moved together with sister kinetochores, the kinetochore fiber stub that remained attached to the kinetochore after the severing, and the intact kinetochore fiber of the sister kinetochore (Fig. 8b) (Kajtez et al. 2016). All these structures moved as a single object away from the spindle, and later back towards the spindle. Thus, the bridging fiber is indeed strongly linked to kinetochore fibers, acting as a bridge between them.

Severing of a kinetochore fiber at different locations revealed that the kinetochore fiber is laterally linked with the bridging fiber in a large region starting ~1 µm away from the kinetochore and extending towards the spindle pole (Milas and Tolic 2016; Kajtez et al. 2016). In the region up to ~1 µm away from the kinetochore, the kinetochore fiber and the bridging fiber are separated, with a distance between them of ~250 nm at the location of the kinetochore (Kajtez et al. 2016).

**Fig. 9:**
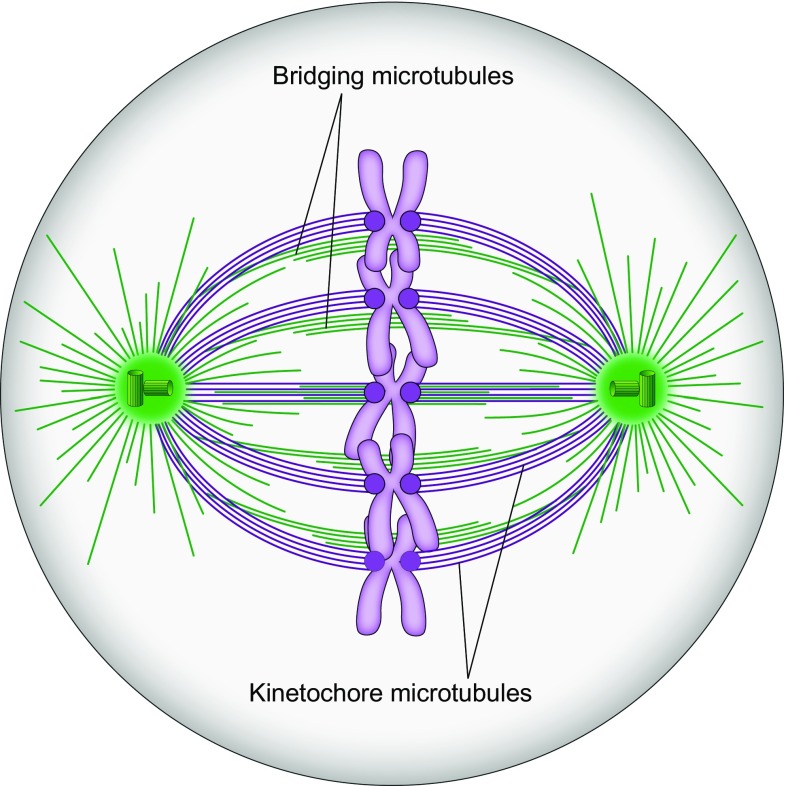
Revised scheme of the spindle. The spindle in a human somatic cell is made of modules consisting of a pair of sister kinetochore fibers and a bridging fiber that connects them. Kinetochore microtubules are shown in purple and all non-kinetochore ones, including bridging microtubules, in *green*. Centrosomes are indicated as *green circles* with *small cylinders* representing centrioles, and chromosomes are *purple* with kinetochores depicted as *dark purple circles*

The bridging fiber consists of 10–15 microtubules arranged in an anti-parallel manner, based on the observation that they bind PRC1 (Fig. 8c), a crosslinking protein localized in the antiparallel overlaps of microtubules in vitro (Bieling et al. 2010; Subramanian et al. 2013; Kapitein et al. 2008) and of the spindle midzone (Jiang et al. 1998; Mollinari et al. 2002; Pellman et al. 1995). By taking advantage of the variable karyotype of HeLa cells, we found that the number of PRC1-decorated bundles per spindle roughly matches the number of kinetochore pairs, indicating a nearly one-to-one relationship between the interpolar bundles and chromosomes (Polak et al. 2017). Localization of the PRC1-labeled bundles with respect to kinetochores showed that more than 90% of PRC1 bundles are associated with a pair of sister kinetochores. Thus, virtually all interpolar bundles in a metaphase spindle are bridging fibers (Fig. 9). In other words, there are practically no “free” overlap bundles in an unperturbed metaphase spindle.

Similarly, more than 90% of kinetochore pairs in a spindle have a bridging fiber connecting their kinetochore fibers in metaphase (Polak et al. 2017). This observation argues against an alternative interpretation in which the non-kinetochore microtubules lying between sister kinetochores are a result of merotelic attachments, where one sister kinetochore is attached to microtubules emanating from both spindle poles (Gregan et al. 2011).

It is still unknown where the plus and minus ends of the bridging microtubules are located. The PRC1-decorated overlap zone of antiparallel microtubules extends roughly 2 µm along each sister k-fiber away from the kinetochore (Kajtez et al. 2016; Polak et al. 2017), which indicates that the plus ends of bridging microtubules are found within that region. Previous electron microscopy experiments have shown that the minus ends of interpolar microtubules are found along kinetochore fibers (Fig. 7e) (Mastronarde et al. 1993). Thus, the minus ends of bridging microtubules are likely found along the kinetochore fibers (Fig. 9). Identification of the sites where the plus and minus ends of bridging microtubules are situated will require further work.

## The bridging fiber balances the tension between sister kinetochores

In addition to the experimental characterization of the microtubules in the spindle, physical models are necessary for quantitative understanding of forces acting in the spindle (Tolic-Norrelykke 2008; Dumont and Mitchison 2009; Tolic-Norrelykke 2010). In most models, the physical linkage between sister kinetochores is described as an elastic connection, which is mediated by centromeric chromatin (Bloom and Joglekar 2010; Burrack and Berman 2012). Thus, a pair of sister kinetochore fibers and their kinetochores are typically represented by two rods connected by an elastic spring (Joglekar and Hunt 2002; Civelekoglu-Scholey et al. 2013; Vladimirou et al. 2013).

**Fig. 10:**
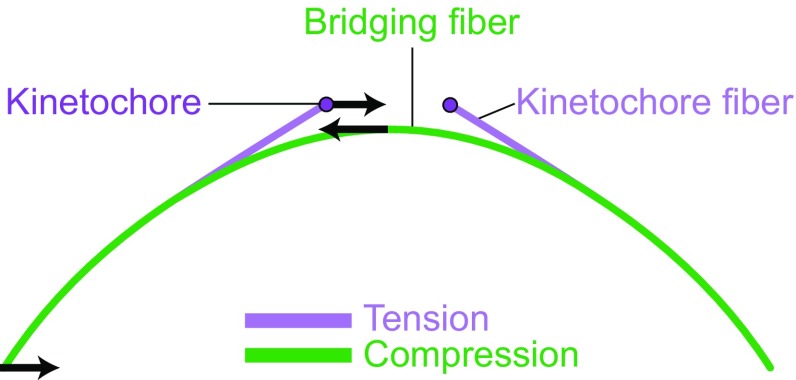
Force balance based on a theoretical model that includes a bridging fiber as a link between sister kinetochore fibers. The compression in the bridging fiber balances the tension on kinetochores and the compression at the spindle pole (*arrows* represent forces). Thus, the bridging fiber allows existence of tension and compression (*purple* and *green* segments, respectively) within an individual kinetochore fiber (Kajtez et al. 2016)

We have recently introduced a physical model that includes the bridging fiber as a link between sister kinetochore fibers (Fig. 10) (Kajtez et al. 2016). In this model, kinetochore fibers and the bridging fiber are elastic rods, which bend under compression. From the observed curvature of the fibers, we assessed the forces acting on them. Our model suggests that the observed shape of the outermost kinetochore fibers can be obtained if a compressive force of ~50 pN acts at the pole, tension of ~300 pN at the kinetochore, and the junction point where the kinetochore fiber merges with the bridging fiber is located ~1 µm away from the kinetochore.

Interestingly, we found that tension and compression coexist along the same kinetochore fiber (Fig. 10) (Kajtez et al. 2016; Tolic and Pavin 2016). The kinetochore fiber is under tension in the region between the kinetochore and the junction point. Conversely, the kinetochore fiber is under compression along the largest part of its length, where the kinetochore fiber is laterally linked with the bridging fiber, i.e., between the junction and the spindle pole. Thus, our model resolves the paradox of the simultaneous existence of tension and compression along a single kinetochore fiber, discussed in (Dumont and Mitchison 2009), by suggesting that the compression in the bridging fiber balances the tension between sister kinetochores and the compression at the spindle pole.

## Open questions

The work on the role of the bridging fibers has opened numerous questions. To what extent is the concept of bridging fibers relevant for other organisms? The findings described here suggest that in mammalian cells the spindle is made of modules consisting of a pair of sister kinetochore fibers and a bundle of interpolar microtubules that connects them. In yeasts, the whole spindle may be thought of as being equivalent to an individual module from a mammalian spindle. Yet, this idea remains to be tested.

How and when is the bridge between sister kinetochore fibers built? In one scenario, kinetochores interact first with an overlap bundle, which will become the bridging fiber after the kinetochore fibers are formed. In the other scenario, kinetochore fibers form first and the bridging fiber is subsequently acquired (Simunic and Tolic 2016). What is the role of the bridging fiber in spindle assembly? Depending on which of the two mentioned scenarios is more applicable to human cells, the bridging fiber may contribute to different extent to the formation of the spindle, congression of the chromosomes to the metaphase plate, establishment of biorientation, setting of the spindle length and maintenance of its structural integrity.

What happens with the bridging fiber during anaphase? If it remains intact and attached to kinetochore fibers, it may contribute to the separation of sister kinetochores. This fiber may serve as a mechanical support for the kinetochore fibers as they shorten and move the kinetochores poleward. Moreover, motor proteins may generate sliding of kinetochore fibers poleward along the bridging fiber. Similarly, microtubules in the bridging fiber may slide apart, pushing the attached kinetochore fibers poleward. It will be exciting to examine these scenarios, both experimentally and theoretically.

Future work will reveal the role of the bridging fiber in critical stages of mitosis including spindle assembly and chromosome segregation. The forces resulting from crosslinking kinetochore fibers and interpolar bundles may emerge as an important part of mitosis.
